# Organellar genome comparisons of *Sargassum polycystum* and *S. plagiophyllum* (Fucales, Phaeophyceae) with other *Sargassum* species

**DOI:** 10.1186/s12864-022-08862-5

**Published:** 2022-09-02

**Authors:** Shuangshuang Zhang, Yanshuo Liang, Jie Zhang, Stefano G. A. Draisma, Delin Duan

**Affiliations:** 1 CAS and Shandong Province Key Laboratory of Experimental Marine Biology, Center for Ocean Mega-Science, Institute of Oceanology, Chinese Academy of Sciences, Qingdao, 266071 China; 2 Laboratory for Marine Biology and Biotechnology, Qingdao National Laboratory for Marine Science and Technology, Qingdao, 266071 China; 3 University of Chinese Academy Sciences, Beijing, 100094 China; 4 Excellence Center for Biodiversity of Peninsular Thailand, Faculty of Science, Prince of Songkla University, Hat Yai, Songkhla, 90110 Thailand

**Keywords:** *Sargassum polycystum*, *Sargassum plagiophyllum*, Mitochondrial genome, Chloroplast genome, Phylogeny

## Abstract

**Background:**

*Sargassum polycystum* C. Agardh and *Sargassum plagiophyllum* C. Agardh are inhabitants of tropical coastal areas, their populations are negatively influenced by global warming and marine environment changes. The mitochondrial and chloroplast genomes of these species have not been sequenced.

**Results:**

The mitochondrial genomes of *S. polycystum* and *S. plagiophyllum* were 34,825 bp and 34,862 bp, respectively, and their corresponding chloroplast genomes were 124,493 bp and 124,536 bp, respectively. The mitochondrial and chloroplast genomes of these species share conserved synteny, sequence regions and gene number when compared with the organellar genomes of other *Sargassum* species. Based on sequence analysis of 35 protein-coding genes, we deduced that *S. polycystum* and *S. plagiophyllum* were closely related with *S. ilicifolium*; these species diverged approximately 0.3 million years ago (Ma; 0.1–0.53 Ma) during the Pleistocene period (0.01–2.59 Ma). Rates of synonymous and non-synonymous substitutions in the mitochondrial genome of the *Sargassum* genus were 3 times higher than those in the chloroplast genome. In the mitochondrial genome, *rpl*5, *rpl*31 and *rps*11 had the highest synonymous substitution rates. In the chloroplast genome, *psa*E, *rpl*14 and *rpl*27 had the highest synonymous substitution rates.

**Conclusions:**

Phylogenetic analysis confirms the close relationship between the two sequenced species and *S. ilicifolium*. Both synonymous and non-synonymous substitution rates show significant divergence between the group of mitochondrial genomes versus the group of chloroplast genomes. The deciphering of complete mitochondrial and chloroplast genomes is significant as it advances our understanding of the evolutionary and phylogenetic relationships between species of brown seaweeds.

**Supplementary Information:**

The online version contains supplementary material available at 10.1186/s12864-022-08862-5.

## Background

*Sargassum polycystum* C. Agardh and *S. plagiophyllum* C. Agardh (Fucales, Phaeophyceae) are large canopy-forming seaweeds that grow in the intertidal and shallow subtidal zones of the tropical Indo-West Pacific, with the former having the widest distribution [1, 2]. With global warming and changes of the marine environment, seaweed forests and ecosystems have recently begun to rapidly diminish [3]. Generally, *Sargassum* species are economically and ecologically important in tropical and temperate coastal areas [2] as they can form seabed forests that play a critical role in the ecology of tropical marine environment [4, 5]. *Sargassum* provides resources for seaweeds fertilizers, alginate, cosmetics, etc., and exhibit antibacterial, antiviral, anti-oxidant [6], and antifungal activities [7].

Deciphering of complete mitochondrial and chloroplast genomes are prerequisites for understanding the evolutionary and phylogenetic background of brown seaweeds [8, 9]. Both mitochondria and chloroplasts are semi-autonomous organelles that preserve their own genetic information [10]. Mitochondria are the main site for respiration and energy supply, whereas chloroplasts are the site of photosynthesis [11]. Generally, organellar genomes reflect the evolutionary history of the algal nuclear genome [12, 13]. Nevertheless, limited information on the organellar genomes of the tropical *Sargassum* species hinders our understanding of their evolutionary history. *Sargassum polycystum* and *S. plagiophyllum* are morphologically distinct; they are considered two different species that occur in sympatry across the *Sargassum* subgenus *Sargassum* section *Polycystae* [14]. Nevertheless, molecular phylogenetic analyses far did not resolve the complexities of the two morphologically different species [15, 16], reflecting their very recent divergence.

Through the sequencing of complete chloroplast and mitochondrial genomes of *S. polycystum* and *S. plagiophyllum*, and by comparing them with those of other *Sargassum* species, we detected variable regions that may be targets for future phylogenetic analysis.

## Results

### Mitochondrial genome characteristics

**Fig. 1:**
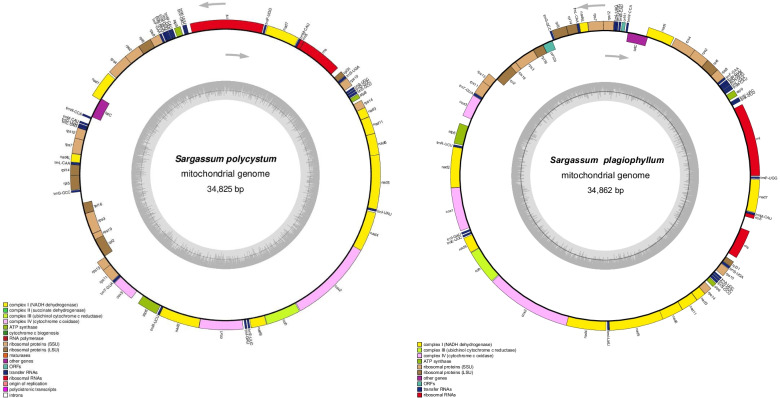
The mitochondrial genome maps of *S. polycystum* and *S. plagiophyllum.* The annotated genes are colored according to the functional categories. Genes on the inside are transcribed in the clock-wise direction, whereas genes on the outside are transcribed in the anticlockwise direction. The ring of bar graphs on the inner circle shows the GC content in dark gray

The mitochondrial genomes (Fig. 1) of *S. polycystum* and *S. plagiophyllum* comprised 34,825 and 34,862 bp, respectively, and were within the size range of previously sequenced mitochondrial genomes (Table 1) of other *Sargassum* species [17] and Stramenopiles [18, 19]. The mitochondrial genome of *S. polycystum* and *S. plagiophyllum* contained 25 tRNA (transfer RNA) genes, 3 rRNA (ribosomal RNA) genes, 37 protein-coding genes (PCGs). In *S. plagiophyllum*, there was an intergenic region located between *rrn5* and *rns*. In *S. plagiophyllum,* only *ORF* (open reading frame) *129, rpl16, rps3, rps19, rpl2* and *tatC* were transcribed from the light strand (L-strand) (clockwise direction in Fig. 1), and the other genes from the complementary heavy strand (H-strand) (anticlockwise in Fig. 1). In *S. polycystum*, the genes of *rpl*16, *rps*3, *rps*19, *rpl*2 and *tat*C were transcribed from the L-strand, and the other genes from the complementary H-strand. In these two *Sargassum* species, the protein-coding regions analogously accounted for 77.50% of the total mitochondrial genome, whereas the non-PCGs regions only accounted for 22.50%. The total GC content (35.71% in *S. polycystum* and 35.70% in *S. plagiophyllum*), the size, gene content and architecture of the mitochondrial genomes were similar to those in other *Sargassum* species (Table 1).

**Table 1:** Genomes features of mitochondria in nine species of the *Sargassum*

Genome Features	*Sargassum polycystum*	*Sargassum plagiophyllum*	*Sargassum thunbergii*	*Sargassum horneri*	*Sargassum fusiforme*	*Sargassum confusum*	*Sargassum hemiphyllum*	*Sargassum muticum*	*Sargassum natans*
Genome Size (bp)/GC Content (%)	34,825/35.71	34,862/35.70	34,748/36.62	34,606/36.16	34,696/37.53	34,721/36.57	34,686/36.57	34,720/36.59	34,727/36.19
Gene number rRNA/tRNA/PCG/Total	3/25/37/65	3/25/37/65	3/25/37/65	3/25/37/65	3/25/37/65	3/25/37/65	3/25/37/65	3/25/37/65	3/25/37/65
PCG Total Length (bp)	26,988	27,024	27,096	27,060	27,117	27,093	27,051	27,069	27,033
PCG Average Length (bp)	729	730	732	731	733	732	731	732	731
PCG’s GC Content (%)	34.65	35.70	35.52	35.13	36.52	35.60	35.58	35.56	35.17
% of Genome (PCGs)	77.50	77.52	77.98	78.19	78.16	78.03	77.99	77.96	77.84
Non-PCGs Length (bp)	7837	7838	7652	7546	7579	7628	7635	7651	7694
% of Genome (Non-PCGs)	22.50	22.48	22.02	21.81	21.84	21.97	22.01	22.04	22.16
GenBank accession	MW485977	MW485978	NC_026700	NC_024613	KJ946428	MG459430	KM210510	KJ938301	NC_033384

### Chloroplast genome characteristics

**Fig. 2:**
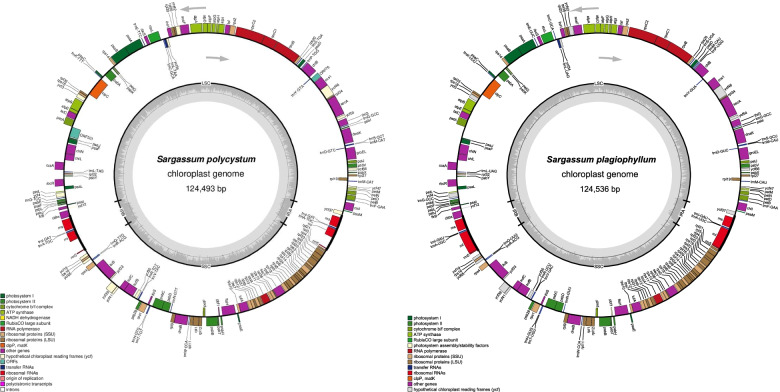
The chloroplast genome maps of *S. polycystum* and *S. plagiophyllum*. The annotated genes are colored according to the functional categories. Genes on the inside are transcribed in the clock-wise direction, whereas genes on the outside are transcribed in the anticlockwise direction. The ring of bar graphs on the inner circle shows the GC content in dark gray

The chloroplast genomes of *S. polycystum* and *S. plagiophyllum* were 124,493 bp and 124,536 bp, respectively, whereas the size of the chloroplast genome in the other *Sargassum* species (namely *S. horneri, S. thunbergii, S. fusiforme, S. confusum* and *S. muticum*) (Table 2) ranged from 124,068 to 124,592 bp. The two assembled chloroplast genomes each had a total of 172 genes, which included 139 PCGs, 27 tRNA genes, and 6 rRNA genes (Fig. 2). The *S. polycystum* chloroplast genome had one 73,674 bp large single-copy (LSC) region, one 39,967 bp small single-copy (SSC) region and a pair of 5426 bp inverted repeats (i.e., IRa and IRb) regions. The chloroplast genome size of *S. plagiophyllum* was 43 bp larger than that of *S. polycystum*, because it had one 73,693 bp LSC region, one 39,971 bp SSC region, and two IR regions of 5436 bp each. Compared to the chloroplast genomes in other *Sargassum* species, the chloroplast genome of *S. plagiophyllum* lost the *trn*L*-*UAA gene and *S. polycystum* lost the *trn*K-UUU gene. The *rnl* gene was 1215 bp in *S. polycystum* and 1698 bp in *S. plagiophyllum*. The chloroplast genomes of *S. polycystum* and *S. plagiophyllum* were otherwise identical in their structure, gene count, and gene content [see Additional files 1 and 2], except for the before mentioned missing *trn*L*-*UAA and *trn*K-UUU genes.

**Table 2:** Genomes features of chloroplasts in seven species of the *Sargassum*

Genome Features	*Sargassum polycystum*	*Sargassum plagiophyllum*	*Sargassum horneri*	*Sargassum thunbergii*	*Sargassum fusiforme*	*Sargassum confusum*	*Sargassum muticum*
Genome Size (bp)/GC Content (%)	124,493/30.42	124,536/30.43	124,068/30.61	124,592/30.40	124,286/30.43	124,375/30.35	124,401/30.40
LSC size (bp)/GC content (%)	73,674/29.02	73,693/29.06	73,311/29.22	73,668/29.01	73,437/29.20	73,552/29.10	73,582/29.22
SSC size (bp)/GC content (%)	39,967/29.56	39,971/29.53	39,885/29.80	40,032/29.52	39,950/29.93	39,941/29.86	39,931/29.99
IR size (bp)/GC content (%)	10,852/43.27	10,872/43.03	10,618/43.77	10,892/43.37	10.898/40.39	10,882/40.42	10,888/40.28
Gene number rRNA/ tRNA/PCG/ Total	6/27/139/172	6/27/139/172	6/28/139/173	6/28/139/173	6/28/139/173	6/28/139/173	6/28/139/173
PCG Total Length (bp)	95,709	93,684	95,751	95,649	95,820	95,826	95,859
PCG Average Length (bp)	689	674	771	770	771	689	690
PCG’s GC Content (%)	30.86	31.01	30.91	30.85	30.82	30.74	30.81
% of Genome (PCGs)	76.88	75.23	77.18	76.77	77.10	77.05	77.06
Non-PCGs Length (bp)	28,784	30,852	28,317	28,943	28,466	28,549	28,542
% of Genome (Non-PCGs)	23.12	24.77	22.82	23.23	22.90	22.95	22.94
GenBank accession	MW485983	MW485981	KP881334	NC_029134	MN121852	MG459429	MW784166

In addition, there were no introns observed in the tRNA regions of the chloroplast genomes of *S. plagiophyllum* and *S. polycystum*, and only one intron was observed in the five other *Sargassum* species compared here. The features of the chloroplast genomes of these two *Sargassum* species and five other *Sargassum* species were summarized in Table 2.

### IR boundary analysis

**Fig. 3:**
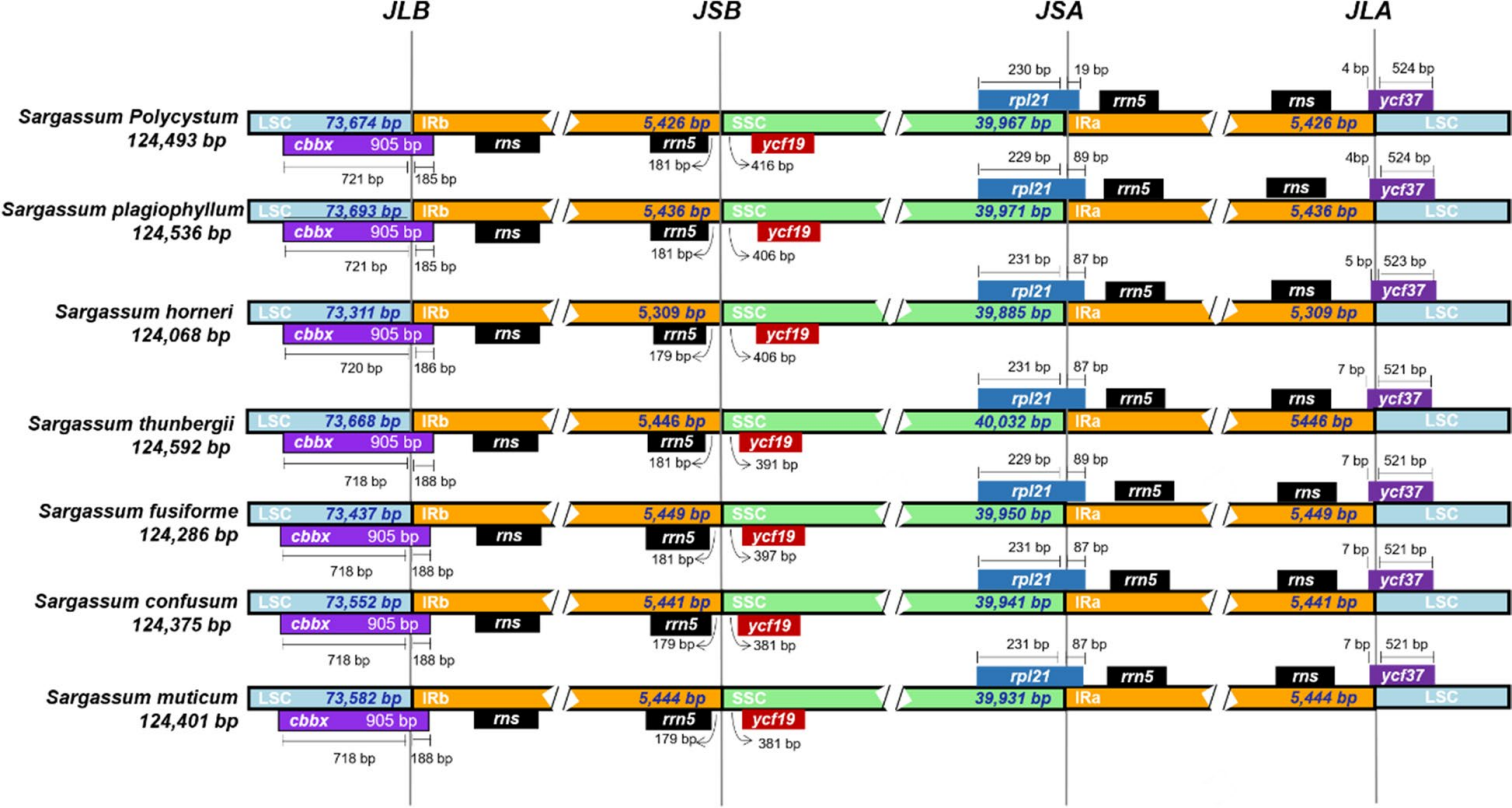
Comparison of Inverted repeat (IR), Large single-copy (LSC) and Small single-copy (SSC) boundary regions in seven *Sargassum* species. JLB: Junction of LSC-IRb, JSB: Junction of SSC-IRb, JSA: Junction of SSC-IRa, JLA: Junction of LSC-IRa

Although the chloroplast genomes of the *Sargassum* species studied were highly conserved, structural variations were prevalent in the IR boundary region (Fig. 3). In *S. polycystum* and *S. plagiophyllum*, the *cbbx* gene spanned the junction between the LSC and IRb regions; this gene extended 721 bp into the LSC and 185 bp into the IRb regions. At the junction of the SSC-IRb (JSB) regions, the *rrn5* gene was located in the IRb region, 181 bp (*S. polycystum* and *S. plagiophyllum*) from the junction; the *ycf*19 gene was located in the SSC region, 416 bp (for *S. polycystum*) and 406 bp (for *S. plagiophyllum*) from the junction. The *rpl*21 gene spanned the junction of the SSC-IRa (JSA) regions, with most of the gene (230 bp for *S. polycystum* and 229 bp for *S. plagiophyllum*) encoded in the SSC region and the remaining (19 bp for *S. polycystum* and 89 bp for *S. plagiophyllum*) in the IRa region. The junction of the LSC-IRa (JLA) regions was spanned by the *ycf*37 gene, 4 bp of which was in the IRa region and 524 bp of which was in the LSC region in both *S. polycystum* and *S. plagiophyllum*; this deviated slightly in other species. The IRa, IRb, LSC and SSC regions of *S. plagiophyllum* were 10 bp, 10 bp, 19 bp and 4 bp longer than those in *S. polycystum*, respectively.

To determine the differences in the chloroplast genomes of *Sargassum* species, we compared the IR/LSC/SSC boundary regions with those in five other *Sargassum* species. We found that they all share the same IR/LSC/SSC regions, with only minor differences in the length of each region (Fig. 3). The IR regions of *S. horneri*, with the shortest chloroplast genome, were 234 bp and 254 bp shorter than *S. polycystum* and *S. plagiophyllum* respectively. Remarkably, the *rpl*21 gene in *S. polycystum* was much shorter compared to the other species and overlapped only by 19 bp with IRa, whereas in the other species it overlapped with IRa by 87–89 bp.

### Substitution rate estimations

We compared the non-synonymous (dN) and synonymous (dS) substitution rates of the sequences of 35 mitochondrial PCGs, excluding *ORF*129 and *ORF*41, [see Additional file 3] and 114 chloroplast PCGs [see Additional file 4] in *S. polycystum* and *S. plagiophyllum*, as well as in five other *Sargassum* species (namely *S. horneri, S. thunbergii, S. fusiforme, S. confusum*, and *S. muticum*). In the 35 mitochondrial PCGs, *rps*11 and *tat*C showed the highest dN (0.0996 and 0.0936, respectively) and moderate dS (0.6769 and 0.4832, respectively) values. *Atp*9 exhibited the lowest dN values with 0.0028. *Nad*4L and *nad*5 had identical dS value (0.4899) and dN value (0.0029), which reflected the similar degree of selection acting upon these two genes among these *Sargassum* species. The dN/dS ratios were different for the 35 PCGs in the mitochondrial genome [see Additional file 5]; the low dN/dS ratios of *nad*4L and *nad*5 (dN/dS = 0.0069) implied their slow evolutionary rate. The higher dN/dS ratio in *tat*C (0.2175) reflected the unique selection process (dN/dS < 0.25) of the 35 PCGs in the mitochondrial genome. Moreover, we verified that the dN/dS ratios of five genes (namely *pet*J, *rpl*21, *psa*D, *rpo*C2 and *thi*S) in the chloroplast genomes were greater than one and that the dN/dS ratios of the remaining genes were lower than one. In the 114 chloroplast PCGs [see Additional file 6], *rpo*C2 and *thi*S exhibited the highest dN/dS ratios with 3.40 and 3.48, respectively, whereas *psb*H, *psb*J, *psb*T, *rpl*29, *atp*H and *pet*D exhibited the lowest and identical dN/dS ratios of 0.001. This implied that these genes were under various degrees of selective pressure.

**Fig. 4:**
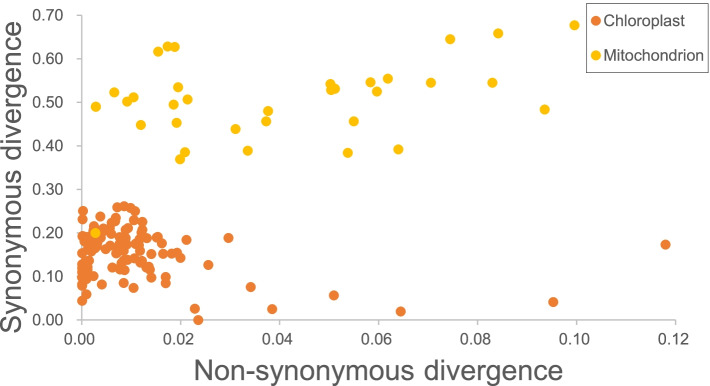
The difference in synonymous and non-synonymous divergence rates between chloroplast and mitochondrial protein-coding genes. The alignments of 114 chloroplast genes (orange) and 35 mitochondrial genes (yellow) from seven *Sargassum* species (five previous reported and two newly sequenced) were used to estimate the divergence values

The dS values of the mitochondrial genes varied from 0.1999 to 0.6769 and those of the chloroplast genes varied from 0.0196 to 0.2615 (Fig. 4). The dN values of the mitochondrial genes varied from 0.0028 to 0.0996 and those of the chloroplast genes varied from 0.0001 to 0.118. Compared with the chloroplast genes, most mitochondrial genes had higher dS and dN values. The average dN and dS values of the mitochondrial PCGs are approximately 3.6 and 3.2 times higher than those of the chloroplast PCGs, respectively. The non-synonymous substitution rates of most PCGs were 0.00–0.02, which accounted for 89.5% of all PCGs from the chloroplast genome.

The average dN/dS ratios of the mitochondrial genes were lower than that of the chloroplast genes (0.08 for the mitochondrion and 0.16 for the chloroplast). The dN/dS ratios of the two organellar genomes from the seven selected *Sargassum* species were less than 0.25.

### Phylogenetic analysis and divergence time of brown algae

**Fig. 5:**
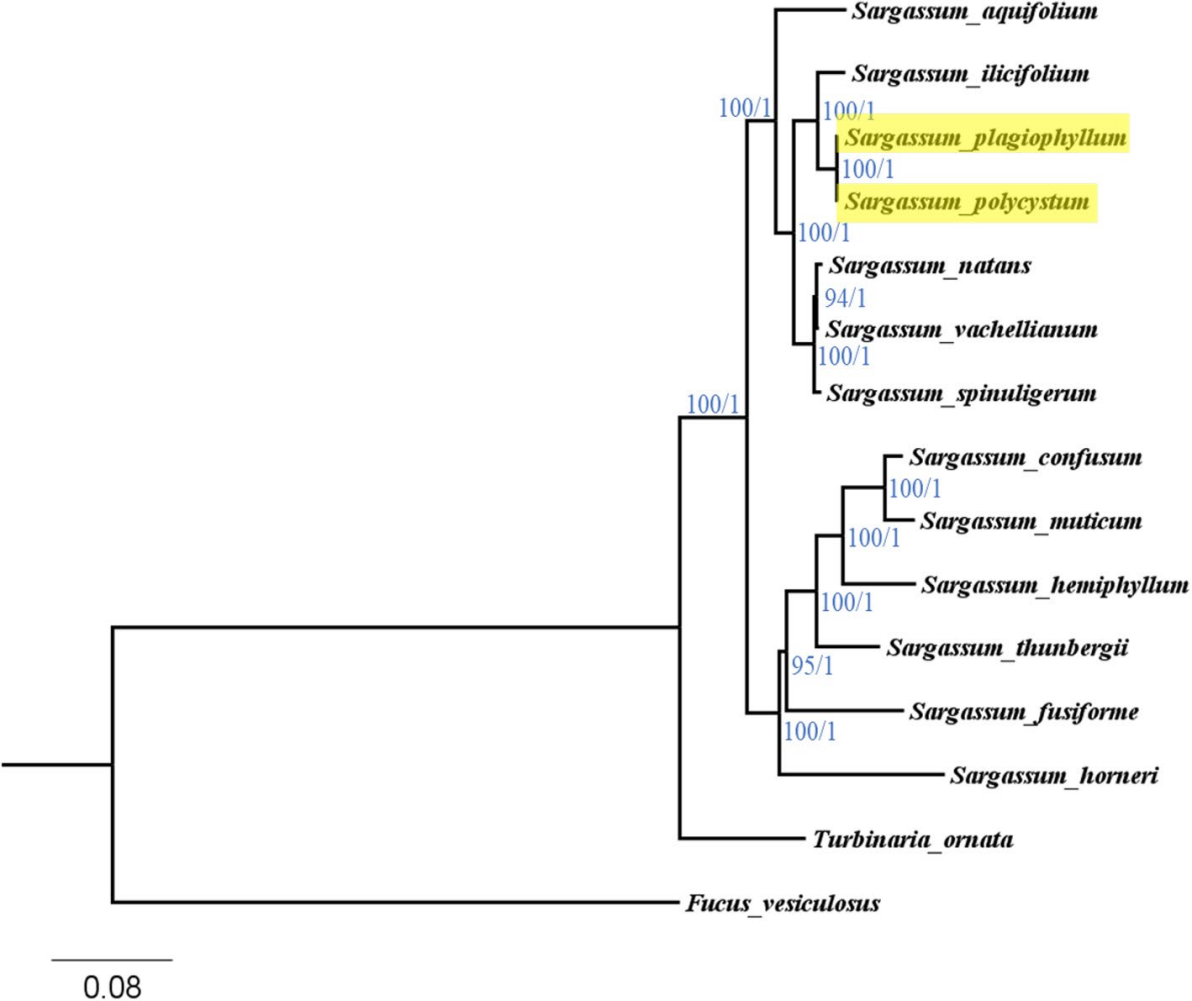
Phylogenetic tree of brown algae constructed using the 35 shared mitochondrial protein-coding genes. The numbers near each node are Maximum likelihood bootstrap support values based on 1000 ultrafast bootstrap replicates in IQ-tree and Bayesian inference posterior probabilities

A 26,360 bp nucleotide alignment was obtained by concatenating 35 shared PCGs from the mitochondrial genomes of thirteen *Sargassum* species, additionally *Turbinaria ornata* (Sargassaceae) and *Fucus vesiculosus* (Fucaceae) were selected as outgroups. This alignment was used to infer phylogenetic relationships between *Sargassum* species using Maximum Likelihood (ML) and Bayesian Inference (BI) approaches. The ML and BI tree were identical in topology; the ML bootstrap support percentages and BI posterior probabilities were shown in Fig. 5. The *Sargassum* clade had maximum support, with infrageneric relationships all having high to maximum support (ML bootstrap ≥94%, BI posterior probability = 1). All *Sargassum* subgenera and sections were monophyletic. The *Polycystae* clade (highlighted in Fig. 5), which consisted of the two species sequenced in this study, was sister to the *Ilicifolia* clade, which only had a single representative in the tree (i.e.*, S. ilicifolium*).

**Fig. 6:**
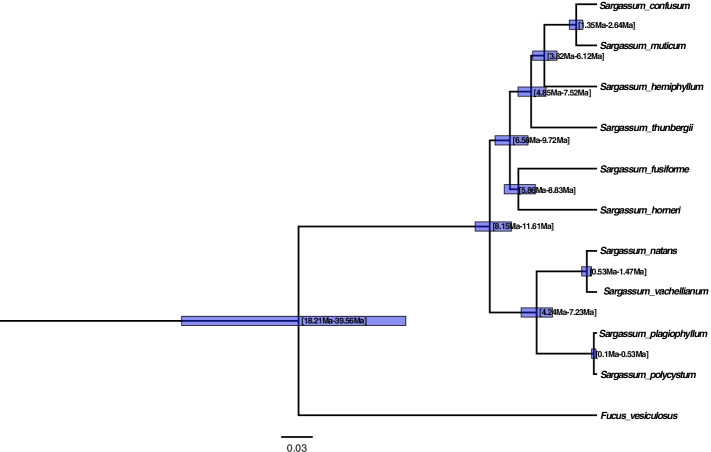
Divergence time estimations of the genus *Sargassum* (Sargassaceae) based on time-calibrated molecular clock analysis. *Fucus vesiculosus* (Fucaceae) is used as the outgroup. Violet bars represent 95% highest posterior density (HPD) intervals of node ages (minimum and maximum values given in square brackets)

We also reconstructed a time tree of *Sargassum* species with divergence times using five mitochondrial genes (*cox*1*, cox*3, *nad*1, *nad*4 and *atp*9) and three chloroplast genes (*rbc*L, *psb*A and *atp*B), with *F. vesiculosus* as the outgroup (Fig. 6). According to this chronogram *S. polycystum* and *S. plagiophyllum* diverged approximately 0.3 Ma (0.1–0.53 Ma), which corresponded with the Pleistocene period (0.01–2.6 Ma). The subgenera *Sargassum* and *Bactrophycus* diverged between 8.2–11.6 Ma (Miocene). The Fucaceae (*F. vesiculosus*) and Sargassaceae (*Sargassum*) diverged between 18.2–39.6 Ma (95% HPD), which corresponded to a geological period spanning early Miocene-Oligocene-late Eocene.

## Discussion

Although the number of rRNA genes and PCGs was identical across the five *Sargassum* species, the *trnL-UAA* and *trnK-UUU* tRNA genes were lost in the chloroplast genomes of *S. plagiophyllum* and *S. polycystum,* respectively. Plastid gene loss has been observed in Dictyochophyceae [20], Eustigmatophyceae [21] and Synurophyceae [22], indicating that gene loss was widespread in Stramenopiles, and these genes were lost during evolution.

The chloroplast genome of *S. plagiophyllum* was 43 bp longer than that of *S. polycystum*. We suspected that this was caused by expansion of the IR regions, which caused the enlargement of the spacing regions between the *trn*A-TGC and *rnl* genes. In *S. plagiophyllum*, IRa and IRb were each an extra 10 bp longer, the LSC region had an extra 19 bp, and the SSC region had an extra 4 bp. The different degrees of IR regions expansion from the chloroplast quadripartite genomes may be related to species diversification.

The dN/dS ratio can be used to estimate the type and degree of selective pressure acting on a protein-coding gene [23]. In our comparison of the organellar genomes data, both synonymous and non-synonymous substitution rates for the mitochondria-encoded genes were more than 3 times higher than those for the chloroplast-encoded genes. The mitochondrial genes in brown algae exhibited a higher mutation rate than the chloroplast genes [24]. Moreover, both *nad*4L and *nad*5 in the mitochondrial genome were involved in the synthesis of Nicotinamide adenine dinucleotide dehydrogenase. They showed similar dN/dS (=0.0069) values, which was possibly driven by their involvement in the same or similar functions. The dN/dS values (dN/dS > 1) of five genes (namely *pet*J, *rpl*21, *psa*D, *rpo*C2 and *thi*S) in the chloroplast genome were considerably higher than the other genes from this organelle, demonstrating that they were under positive selection.

Phylogenetically, *S. plagiophyllum* and *S. polycystum* were sister-species and exhibited very limited genetic divergence and a relatively recent divergence time (0.1–0.53 Ma, 95% HPD). The two species retained their morphological distinctness, yet Stankovic et al. [1] reported that sympatric populations of the two species in Phuket did grow adjacent to each other; *S. plagiophyllum* grows in the low intertidal zones, and *S. polycystum* grows in deeper area of low intertidal zones. In the present study, *S. plagiophyllum* and *S. polycystum* were the closest related sister-taxa with the least genetic divergence. While *S. fusiforme* and *S. horneri*, which grow in moderate seawater temperature conditions [25], were observed to be the most divergent sister-taxa (5.86–8.83 Ma, 95% HPD). Comparatively *S. plagiophyllum* and *S. polycystum*, which grow in tropical temperature conditions [26], are the most closely related clades (diverging 0.1–0.53 Ma, 95% HPD). Decoding of the organellar genomes of *S. plagiophyllum* and *S. polycystum* could enhanced our understanding of the evolution of the *Sargassum* genus.

## Conclusions

We firstly reported for the first time the mitochondrial and chloroplast genomes of *S. plagiophyllum* and *S. polycystum*, describing their genome size and structure. Phylogenetic analysis confimed their close relationship to each other, and to *S. ilicifolium* in the sister-section *Ilicifolia*. Both synonymous and non-synonymous substitution rates reflected significant differences in the selective pressure acting on the mitochondrial and chloroplast genomes. Our study improves our understanding of the phylogenetic and evolutionary history of *Sargassum*.

## Methods

### Algal sample collection and DNA extraction

*Sargassum polycystum* and *S. plagiophyllum* samples were collected from Koh Kut, Trat, Thailand (11°36′34″N 102°35′47″E, Gulf of Thailand) and Penang, Malaysia (5°28′56″N 100°15′18″E, Strait of Malacca), respectively, in 2019 and stored in silica. After sample collection, the total algal DNA was extracted from young blades using the FastPure Plant DNA Isolation Mini Kit (Vazyme Biotech Co., Ltd., Nanjing, China) according to the manufacturer’s instructions. The extracted DNA was subsequently purified according to the DNA Purification Kit (Vazyme Biotech Co., Ltd., Nanjing, China) instructions.

### Genome sequencing, assembly, and annotation

To obtain a full-length mitochondrial and chloroplast sequence, we used the Illumina strategy: For each species of mitochondrion and chloroplast, 1 μg of purified DNA was used to construct a paired-end library. DNA was purified using the TIANgel Midi Purification Kit (Tiangen Biotech Co., Ltd., Beijing, China) following manufacturer’s instructions; purified DNA was subsequently sonicated to ~ 450-bp insert sequences using the Covaris M220 system with the parameter to 450-bp. Sequencing libraries were constructed using the NEBNext Ultra™ DNA Library Prep Kit for Illumina (New England Biolabs, Ipswich, MA, England) following the manufacturer’s instructions. The constructed libraries were sequenced using Illumina NovaSeq 6000 (Biozeron, Shanghai, China). We quality-controlled and trimmed low-quality raw sequences using Trimmomatic-0.39 [27] with the parameters “SLIDINGWINDOW: 4:15 MINLEN: 75”. All the clean reads were used to generate de novo assemblies using SOAP denovo v2.04 [28]. Potential contigs were extracted by aligning against the protein-coding genes from the plant chloroplast database (http://ftp.ncbi.nih.gov/refseq/release/plastid/) and the mitogenome database (http://ftp.ncbi.nlm.nih.gov/refseq/release/mitochondrion/) with BLAST v 2.8.1+ [29]. GapCloser v1.12 [28] was employed to fill the gaps in the scaffolds. Finally, the mitochondrial and chloroplast genomes of *S. polycystum* and *S. plagiophyllum* were obtained.

We used the online Dual Organellar GenoMe Annotator tool with default parameters [30] for annotating PCGs and ORFs. The cloverleaf structures of tRNA were predicted using tRNAscan-SE v1.23 with default parameters [31], and rRNA genes were predicted using RNAmmer v1.2 [32]. The circular physical maps of mitochondrial and chloroplast genomes were generated using OGDRAW v1.3.1 [33]. MEGA 7.0 [34] was used to align the sequences and determine the base composition. Functional annotations were performed using sequence-similarity Blast searches with a typical cut-off E-value of 10^− 5^ [35] against several publicly available protein databases, i.e.*,* NCBI non-redundant (Nr) protein database, Swiss-Prot, Clusters of Orthologous Groups (COGs) and Kyoto Encyclopedia of Genes and Genomes (KEGG) and Gene Ontology (GO). The mitochondria and chloroplast genomes of the two *Sargassum* species were deposited into the GenBank database, with the accession numbers of MW485977 and MW485983 (respectively) for *S. polycystum*, MW485978 and MW485981 for *S. plagiophyllum*.

### Chloroplast genome boundary region analysis

To investigate the contraction and expansion of inverted repeat (IR) boundary regions in the chloroplast genomes of *Sargassum*, we downloaded the chloroplast genome data of *S. horneri*, *S. thunbergii*, *S. fusiforme*, *S. confusum* and *S. muticum* from the GenBank database (Table 2) and compared and visualized the IR boundary positions and their adjacent genes using the IR scope online program [36]. The genome length, GC content, and gene length of chloroplast genome data of all *Sargassum* species were compared using MEGA v7.0 and BioEdit software [37].

### Substitution rate estimation

We compared sequence divergence rates of genes between the mitochondrial and chloroplast genomes of *S. polycystum* and *S. plagiophyllum*, and with other studied *Sargassum* species (namely *S. horneri, S. thunbergii*, *S. fusiforme*, *S. confusum* and *S. muticum*). The non-synonymous (dN) and synonymous (dS) substitution rates for the 35 mitochondrial PCGs and 114 chloroplast PCGs were analyzed. Codon alignments for each PCG were performed with MEGA and the identified conserved blocks of multiple sequence alignments were extracted using Gblocks v0.91b with default parameters [38]. The sequence data were aligned and converted into PML format using DAMBE5 [39]. Subsequently, dN, dS, and the dN/dS ratios were calculated using the CodeML program from the Phylogenetic Analysis by Maximum Likelihood (PAML) package v4.8 [40]. The model was run with the following settings in the codeml.ctl files: runmode = − 2 and CodonFreq = 2. To ensure the accuracy and reliability of the data, synonymous substitution values > 5 were discarded from the subsequent analysis [41]. Bar plots and scatter plots of the values of dN, dS and dN/dS were generated for comparing mutation rates and screening of the selected genes.

### Phylogenetic analysis and divergence time estimation

Phylogenetic relationships were analyzed on the basis of the 35 mitochondrial PCGs (*rps*2–4, *rps*7, *rps*8, *rps*10–14, *rps*19; *rpl*2, *rpl*5, *rpl*6, *rpl*14, *rpl*16, *rpl*31; *nad*1–7, *nad*9, *nad*11; *cob*; *cox*1–3; *atp*6, *atp*8, *atp*9; and *tat*C) for a total of 15 species in the Fucales. *Turbinaria ornata* (Sargassaceae) and *Fucus vesiculosus* (Fucaceae) (GenBank accessions: KM501562 and NC_007683) were used as outgroup taxa. The nucleotide sequences of each gene were aligned using the default setting of ClustalW v2.0 [42]. FASTA [43] was applied to concatenate these sequences. Using IQ-TREEv1.6.11 [44] and MrBayes v3.2 [45], we reconstructed maximum likelihood (ML) and Bayesian inference (BI) trees, respectively. One optimal fit model selection of amino acid replacement was performed using IQ-TREEv1.6.11 [46]. Based on the Bayesian information criterion, GTR + F + I + G4 (I = 0.4387, G = 1.113) was chosen as the optimized model for ML methods. The node support values were estimated in IQ-tree based on 1000 ultrafast bootstrap replicates with other parameters set as default. For the BI tree, Modeltest v3.7 [47] was used for identifying the best-fit substitution model for the concatenated dataset (GTR + G + I, I = 0.4378, G = 1.1099) under the Akaike information criterion. The Markov chain Monte Carlo (MCMC) algorithm parameters were set to: 1 × 10^6^ generations running with four chains (one cold chain and three heated chains) with a tree sampling frequency of every 1000 generations. The results of the first 25% runs were discarded. We used FigTree v1.4.4 (https://tree.bio.ed.ac.uk/softw are/figtree/) to visualize the derived BI and ML trees.

To build the divergence time estimate tree, five mitochondrial genes (*cox*1, *cox*3, *nad*1, *nad*4 and *atp*9) and three chloroplast genes (*rbc*L, *psb*A and *atp*B) from ten *Sargassum* species (representing two subgenera and seven sections) and *Fucus vesiculosus* were concatenated and aligned. The multi-gene alignment was partitioned into 1st, 2nd and 3rd codon positions using DAMBE5 [39]. The ML trees were reconstructed using PhyML v.3.1 under the optimal model of TIM3 + F + I + G4 with 100 bootstrap replicates. MCMCTree of PAML v4.8 [48] was used to estimate the divergence times between ten *Sargassum* species by using the approximate likelihood calculations and two fossil calibrations. We set a minimum age of 13 Ma for the divergence time of Sargassaceae and Fucaceae [49, 50] and the minimum age of the genus *Sargassum* at 6.7 (3.4–11) Ma based on the divergence time estimate of the Sargassacean genera *Turbinaria* and *Sargassum* in Yip et al. [14]. The overall substitution rates of the ML tree acquired through BASEML in PML were measured before rgene-gamma calculations. The gradient and Hessian of the branch lengths were estimated by BASEML using the TIM3 + F + I + G4 substitution model at the maximum likelihood estimates [51]. The gradient was calculated using a different method, and the Hessian parameter was adopted for calculating the estimated scores [52, 53]. We used the program MCMCTree to estimate the divergence times under both the independent rates model (clock = 2 in the mcmctree.ctl control file) and the nucleotide substitution model (TIM3 + F + I + G4). The substitution rate per time unit (0.080406), rgene_gamma (1 12.5) and sigma2_gamma (1 4.5) were used as parameters. To determine whether convergence had been achieved, two independent MCMC chains were run with 5 × 10^6^ steps after discarding 10^4^ generations as burn-in.

## Supplementary Information

**Additional file 1: Table S1.** The chloroplast genome coding genes of *Sargassum polycystum*.

**Additional file 2: Table S2.** The chloroplast genome coding genes of *Sargassum plagiophyllum*.

**Additional file 3: Table S3.** The dN/dS ratio, dN and dS values of 35 mitochondrial PCGs from 7 brown algae.

**Additional file 4: Table S4.** The dN/dS ratio, dN and dS values of 114 chloroplast genes from 7 brown algae.

**Additional file 5: Fig. S1.** The dN/dS of mitochondrial genes (*n* = 35) estimated from the *Sargassum* species. The ratio of non-synonymous and synonymous sequence divergence to the 7 species of brown algae.

**Additional file 6: Fig. S2.** The dN/dS for chloroplast genes (*n* = 114) estimated to the *Sargassum* species. Shown is the ratio of non-synonymous and synonymous sequence divergence to the 7 species with scales in top and bottom panel.

## Data Availability

All datasets supporting the conclusions of this article are included within the article and its additional files. The datasets used and/or analyzed during this study are available from the corresponding author on reasonable request, and the GenBank accession numbers of the mitochondrial genomes of *S. plagiophyllum* and *S. polycystum* are MW485978 and MW485977, respectively, and the chloroplast genomes of *S. plagiophyllum* and *S. polycystum* are MW485981 and MW485983, respectively. The raw reads of *S. plagiophyllum* and *S. polycystum* organelle genomes have been deposited in the NCBI Sequence Read Archive under the BioProject numbers PRJNA851243 and PRJNA851213, respectively, Sequence Read Archive accession number of *S. plagiophyllum* and *S. polycystum* are SAMN29219088 and SAMN29214723 respectively.

## Abbreviations

Ma: megaannus (Million Years Ago)
ORFs: Open Reading Frames
PCGs: Protein-coding genes
LSC: Large single-copy
SSC: Small single-copy
IR: Inverted repeat
JLB: Junction of LSC-IRb
JSB: Junction of SSC-IRb
JSA: Junction of SSC-IRa
JLA: Junction of LSC-IRa
dN: Non-synonymous substitution
dS: Synonymous substitution
HPD: Highest posterior density
ML: Maximum likelihood
BI: Bayesian inference
tRNA: Transfer RNA
rRNA: Ribosomal RNA
MCMC: Markov chain Monte Carlo
