# Supplementary material for: Organellar genome comparisons of Sargassum polycystum and S. plagiophyllum (Fucales, Phaeophyceae) with other Sargassum species
Source: BMC Genomics. 2022 Sep 2;23:629. doi: 10.1186/s12864-022-08862-5 (PMC9438170; doi:10.1186/s12864-022-08862-5)
Supplement: Supplementary file 1 — Additional file 1: Table S1. The chloroplast genome coding genes of Sargassum polycystum. [file 12864_2022_8862_MOESM1_ESM.pdf]

Table S1 The chloroplast genome coding genes of *Sargassum polycystum*

Classification	Gene function type	Number	Gene name
Photosynthesis	Photosystem Ⅰ	12	*psaA*, *psaB*, *psaC*, *psaD*, *psaE*, *psaF*, *psaI*, *psaJ*, *psaL*, *psaM*, *ycf3*, *ycf4*
	Photosystem II	18	*pbf1*, *psbA*, *psbB*, *psbC*, *psbD*, *psbE*, *psbF*, *psbH*, *psbI*, *psbJ*, *psbK*, *psbL*, *psbT*, *psbV*, *psbX*, *psbY*, *psb28*, *ycf12*
	Electron transport and ATP synthesis	19	*petA*, *petB*, *petD*, *petF*, *petG*, *petJ*, *petL*, *petM*, *petN*, *ccsA*, *ccs1*, *atpA*, *atpB*, *atpD*, *atpE*, *atpF*, *atpG*, *atpH*, *atpI*
	Carbon assimilation	6	*rbcL*, *rbcS*, *ilvB*, *ilvH*, *thiG*, *thiS*
	Light harvesting and chl Biosynthesis	5	*ascF*, *chlI*, *chlL*, *chlN*, *chlB*
Self-replication	Transcription and Translation	52	*rpl1*, *rpl2*, *rpl3*, *rpl4*, *rpl5*, *rpl6, rpl9*, *rpl11*, *rpl12*, *rpl13*, *rpl14*, *rpl16*, *rpl18*, *rpl19*, *rpl20*, *rpl21*, *rpl22*, *rpl23*, *rpl24*, *rpl27*, *rpl29*, *rpl31*, *rpl32*, *rpl33*, *rpl34*, *rpl35*, *rpl36*, *rps1*, *rps2*, *rps3*, *rps4*, *rps5*, *rps7*, *rps8*, *rps9*, *rps10*, *rps11*, *rps12*, *rps13*, *rps14*, *rps16*, *rps17*, *rps18*, *rps19*, *rps20*, *rpoA*, *rpoB*, *rpoC1*, *rpoC2*, *dnaB*, *tsf*, *tufA*
	tRNA	27	*trnA-TGC(1)*, *trnA-TGC(2)*, *trnA-GTC*, *trnC-GCA*, *trnD-GTC*, *trnE-TTC*, *trnF-GAA*, *trnG-TCC*, *trnG-GCC*, *trnH-GTG*, *trnI-GAT*, *trnL-TAA*, *trnL-TAG*, *trnM-CAT(1)*, *trnM-CAT(2)*, *trnN-GTT*, *trnP-TGG*, *trnP-GAA*, *trnQ-TTG*, *trnR-ACG*, *trnR-TCT*, *trnS-TGA*, *trnS-GCT*, *trnT-TGT*, *trnV-TAC*, *trnW-CCA*, *trnY-GTA*
	rRNA	6	*rns-1*, *rns-2*, *rnl-1*, *rnl-2*, *rrn5-1*, *rrn5-2*
Other genes	Signal transduction	3	*cbbX*, *ftrB*, *rbcR*
	Protein import	3	*tatC*, *secA*, *secY*
	Fe-S assembly	2	*sufB*, *sufC*
	Chaperones	2	*dnaK*, *groEL*
	Proteolysis	2	*clpC*, *ftsH*
	Conserved hypothetical genes	15	*orf76*, *orf501*, *ycf19*, *ycf33*, *ycf34*, *ycf35*, *ycf37*, *ycf39*, *ycf41*, *ycf42*, *ycf46*, *ycf47*, *ycf54*, *ycf65*, *ycf66*
