# Supplementary material for: Organellar genome comparisons of Sargassum polycystum and S. plagiophyllum (Fucales, Phaeophyceae) with other Sargassum species
Source: BMC Genomics. 2022 Sep 2;23:629. doi: 10.1186/s12864-022-08862-5 (PMC9438170; doi:10.1186/s12864-022-08862-5)
Supplement: Supplementary file 3 — Additional file 3: Table S3. The dN/dS ratio, dN and dS values of 35 mitochondrial PCGs from 7 brown algae. [file 12864_2022_8862_MOESM3_ESM.pdf]

**Table S3** The dN/dS ratio, dN and dS values of 35 mitochondrial PCGs from 7 brown algae

| Gene name | dN/dS | dN | dS |
|---|---|---|---|
| *atp*6 | 0.0268 | 0.0120 | 0.4478 |
| *atp*8 | 0.0618 | 0.0199 | 0.3693 |
| *atp*9 | 0.0150 | 0.0028 | 0.1999 |
| *cob* | 0.0377 | 0.0188 | 0.6273 |
| *cox*1 | 0.0202 | 0.0105 | 0.5117 |
| *cox*2 | 0.1689 | 0.083 | 0.5449 |
| *cox*3 | 0.0267 | 0.0174 | 0.6283 |
| *nad*1 | 0.0127 | 0.0066 | 0.5226 |
| *nad*2 | 0.0526 | 0.0214 | 0.5064 |
| *nad*3 | 0.0201 | 0.0092 | 0.5016 |
| *nad*4 | 0.0477 | 0.0195 | 0.5347 |
| *nad*4L | 0.0069 | 0.0029 | 0.4899 |
| *nad*5 | 0.0069 | 0.0029 | 0.4899 |
| *nad*6 | 0.1352 | 0.0706 | 0.5448 |
| *nad*7 | 0.0276 | 0.0155 | 0.6164 |
| *nad*9 | 0.0439 | 0.0192 | 0.4528 |
| *nad*11 | 0.0427 | 0.0186 | 0.4945 |
| *rpl*2 | 0.0989 | 0.0512 | 0.5312 |
| *rpl*5 | 0.1093 | 0.0745 | 0.6450 |
| *rpl*6 | 0.1107 | 0.0584 | 0.5459 |
| *rpl*14 | 0.0825 | 0.0373 | 0.4562 |
| *rpl*16 | 0.1102 | 0.0597 | 0.5246 |
| *rpl*31 | 0.1354 | 0.0842 | 0.6580 |
| *rps*2 | 0.1082 | 0.0619 | 0.5543 |
| *rps*3 | 0.0911 | 0.0503 | 0.5420 |
| *rps*4 | 0.0744 | 0.0377 | 0.4798 |
| *rps*7 | 0.1243 | 0.0550 | 0.4562 |
| *rps*8 | 0.1507 | 0.0311 | 0.4386 |
| *rps*10 | 0.1790 | 0.0640 | 0.3919 |
| *rps*11 | 0.1522 | 0.0996 | 0.6769 |
| *rps*12 | 0.0887 | 0.0209 | 0.3855 |
| *rps*13 | 0.1286 | 0.0538 | 0.3839 |
| *rps*14 | 0.0984 | 0.0504 | 0.5284 |
| *rps*19 | 0.0085 | 0.0336 | 0.3889 |
| *tat*C | 0.2175 | 0.0936 | 0.4832 |
