# Supplementary material for: Organellar genome comparisons of Sargassum polycystum and S. plagiophyllum (Fucales, Phaeophyceae) with other Sargassum species
Source: BMC Genomics. 2022 Sep 2;23:629. doi: 10.1186/s12864-022-08862-5 (PMC9438170; doi:10.1186/s12864-022-08862-5)
Supplement: Supplementary file 4 — Additional file 4: Table S4. The dN/dS ratio, dN and dS values of 114 chloroplast genes from 7 brown algae. [file 12864_2022_8862_MOESM4_ESM.pdf]

Table S4 The dN/dS ratio, dN and dS values of 114 chloroplast genes from 7 brown algae

Gene name	dN/dS	dN	dS
*atp*A	0.0045	0.0006	0.1806
*atp*B	0.0194	0.0038	0.2378
*atp*D	0.0559	0.0106	0.2294
*atp*E	0.0330	0.0012	0.1155
*atp*F	0.0913	0.0122	0.1358
*atp*G	0.0468	0.0070	0.1528
*atp*H	0.0010	0.0002	0.1920
*atp*I	0.0705	0.0085	0.1588
cbbx	0.0115	0.0020	0.1572
*ccs*A	0.0661	0.0077	0.1752
*chl*B	0.0388	0.0032	0.1778
*chl*I	0.0372	0.0049	0.1629
*dna*B	0.1501	0.0200	0.1426
*dna*K	0.0304	0.0057	0.2105
*ftr*B	0.0644	0.0076	0.1186
*fts*H	0.0100	0.0015	0.1362
*gro*EL	0.0119	0.0025	0.2153
*ilv*H	0.1000	0.0131	0.1205
*pet*A	0.0550	0.0123	0.2069
*pet*B	0.0089	0.0013	0.1637
*pet*D	0.0010	0.0003	0.2504
*pet*F	0.1373	0.0212	0.1842
*pet*J	1.3022	0.0510	0.0562
*pet*L	0.0735	0.0060	0.1977
*pet*M	0.0051	0.0001	0.0441
*pet*N	0.0222	0.0001	0.1192
*psa*A	0.0162	0.0023	0.1702
*psa*B	0.0101	0.0024	0.2055
*psa*C	0.0475	0.0039	0.1891
*psa*D	1.6494	0.0229	0.0261
*psa*E	0.0470	0.0099	0.2572
*psa*M	0.0250	0.0002	0.1927
*psb*B	0.0043	0.0008	0.1909
*psb*C	0.0093	0.0012	0.1217
*psb*D	0.0061	0.0008	0.1352
*psb*E	0.0221	0.0002	0.2314
*psb*F	0.0225	0.001	0.0588
*psb*H	0.0010	0.0001	0.0978
*psb*I	0.0209	0.0001	0.1532
*psb*J	0.0010	0.0001	0.1276

*psb*L	0.0198	0.0001	0.1098
*psb*T	0.0010	0.0001	0.0788
*psb*V	0.1746	0.0105	0.0737
*psb*X	0.1219	0.0085	0.0852
*psb*28	0.0479	0.0108	0.2502
*rbc*L	0.0121	0.0016	0.1605
*rbc*R	0.0459	0.0091	0.1731
*rbc*S	0.0474	0.0089	0.1886
*rpl*1	0.1197	0.0138	0.1155
*rpl*2	0.0533	0.0123	0.2251
*rpl*3	0.1400	0.0182	0.1523
*rpl*4	0.1271	0.0193	0.1542
*rpl*5	0.0699	0.0109	0.1744
*rpl*6	0.0763	0.0132	0.1880
*rpl*9	0.2093	0.0236	0.12490.
*rpl*11	0.0477	0.0089	0.2070
*rpl*12	0.2316	0.0170	0.0847
*rpl*13	0.0716	0.0154	0.1907
*rpl*14	0.0284	0.0072	0.2587
*rpl*16	0.0494	0.0087	0.1811
*rpl*18	0.1708	0.0081	0.1310
*rpl*20	0.0770	0.0117	0.1856
*rpl*21	1.5856	0.0385	0.0251
*rpl*22	0.0645	0.0121	0.2008
*rpl*23	0.0969	0.0093	0.1384
*rpl*24	0.2383	0.0141	0.0974
*rpl*27	0.0459	0.0086	0.2615
*rpl*29	0.001	0.0001	0.0980
*rpl*31	0.0780	0.0041	0.0815
*rpl*33	0.2611	0.0297	0.1884
*rpl*34	0.0834	0.0110	0.1755
*rpl*35	0.2441	0.0170	0.0987
*rpl*36	0.0032	0.0001	0.0820
*rpo*A	0.1150	0.0165	0.1519
*rpo*B	0.0502	0.0088	0.1824
*rpo*C2	3.4004	0.0645	0.0196
*rps*1	0.0752	0.0087	0.1147
*rps*2	0.0331	0.0058	0.1707
*rps*3	0.0194	0.0039	0.2012
*rps*4	0.1416	0.0136	0.1222
*rps*5	0.0559	0.0106	0.2294
*rps*7	0.0201	0.0031	0.1691
*rps*8	0.0201	0.0010	0.0941

*rps*9	0.0288	0.0069	0.2346
*rps*10	0.0672	0.0094	0.2109
*rps*11	0.0295	0.0061	0.2235
*rps*12	0.0078	0.0010	0.1288
*rps*13	0.0682	0.0078	0.1160
*rps*14	0.1377	0.0119	0.1317
*rps*16	0.0494	0.0085	0.1369
*rps*17	0.0874	0.0141	0.1515
*rps*18	0.0603	0.0077	0.1904
*rps*19	0.0122	0.0022	0.1840
*rps*20	0.4663	0.0342	0.0755
*sec*A	0.0431	0.0078	0.1691
*sec*Y	0.0742	0.0107	0.1418
*suf*B	0.019	0.0044	0.2039
*tat*C	0.0639	0.0116	0.1717
*thi*G	0.0784	0.0118	0.1591
*thi*S	3.4770	0.0953	0.0413
tsf	0.0865	0.1180	0.1729
*tuf*A	0.0096	0.0019	0.1935
*ycf*3	0.0127	0.0028	0.1907
*ycf*4	0.0696	0.0068	0.2266
*ycf*19	0.0442	0.0074	0.1606
*ycf*35	0.1008	0.0162	0.1760
*ycf*39	0.0682	0.0076	0.1692
*ycf*41	0.2140	0.0256	0.1263
*ycf*42	0.0214	0.0044	0.2099
*ycf*46	0.0151	0.0027	0.1648
*ycf*47	0.0729	0.0062	0.1204
*ycf*54	0.0536	0.0072	0.1552
*ycf*65	0.1056	0.0152	0.1896
*ycf*66	0.0534	0.0024	0.1006
