# Supplementary material for: Organellar genome comparisons of Sargassum polycystum and S. plagiophyllum (Fucales, Phaeophyceae) with other Sargassum species
Source: BMC Genomics. 2022 Sep 2;23:629. doi: 10.1186/s12864-022-08862-5 (PMC9438170; doi:10.1186/s12864-022-08862-5)
Supplement: Supplementary file 5 — Additional file 5: Fig. S1. The dN/dS of mitochondrial genes (n = 35) estimated from the Sargassum species. The ratio of non-synonymous and synonymous sequence divergence to the 7 species of brown algae. [file 12864_2022_8862_MOESM5_ESM.pdf]

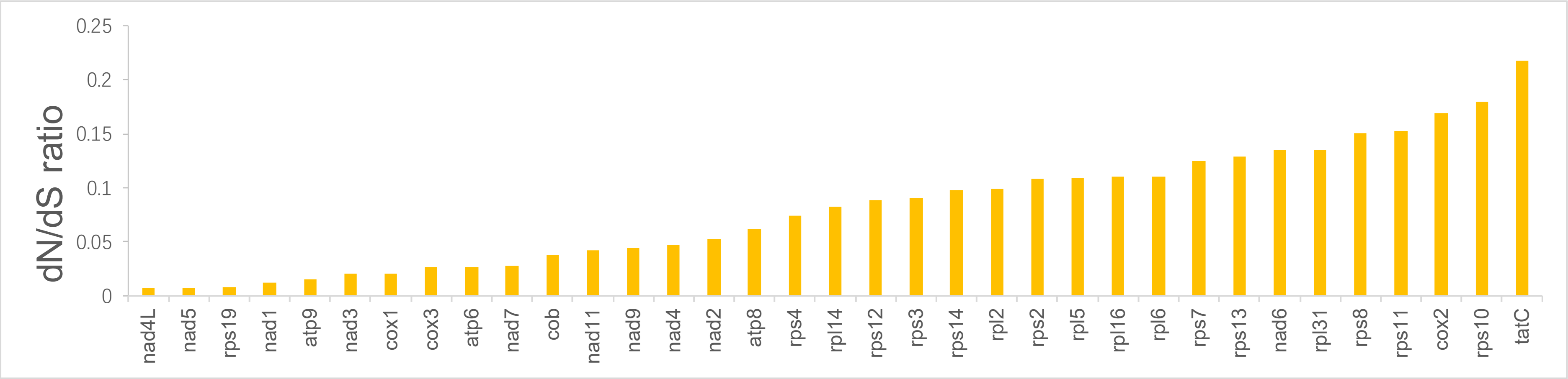

Figure S1 The dN/dS of mitochondrial genes (n = 35) estimated from the *Sargassum* species

The ratio of non-synonymous and synonymous sequence divergence to the 7 species of brown algae.
