# Supplementary material for: Organellar genome comparisons of Sargassum polycystum and S. plagiophyllum (Fucales, Phaeophyceae) with other Sargassum species
Source: BMC Genomics. 2022 Sep 2;23:629. doi: 10.1186/s12864-022-08862-5 (PMC9438170; doi:10.1186/s12864-022-08862-5)
Supplement: Supplementary file 6 — Additional file 6: Fig. S2. The dN/dS for chloroplast genes (n = 114) estimated to the Sargassum species. Shown is the ratio of non-synonymous and synonymous sequence divergence to the 7 species with scales in top and bottom panel. [file 12864_2022_8862_MOESM6_ESM.pdf]

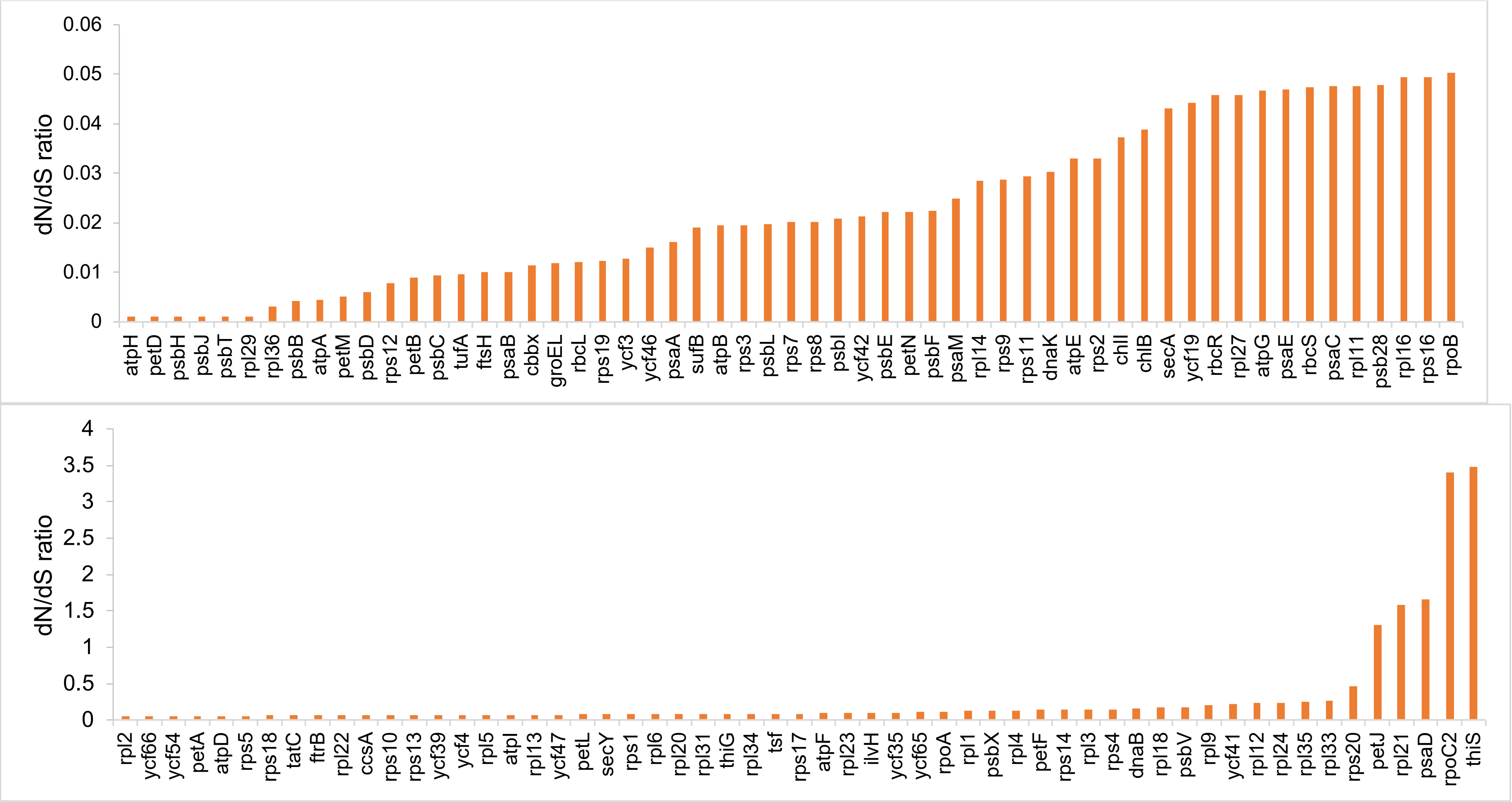

Figure S2 The dN/dS for chloroplast genes (n = 114) estimated to the *Sargassum* species
Shown is the ratio of non-synonymous and synonymous sequence divergence to the 7 species with scales in top and bottom panel.
